# Endoscopic closure of a rectovaginal fistula following surgery for endometriosis using the MARCEAU system

**DOI:** 10.1055/a-2155-3708

**Published:** 2023-09-01

**Authors:** Elena De Cristofaro, Pierre Lafeuille, Louis-Jean Masgnaux, Olivier Rouquette, Anne-Sophie Grémeau, Jérôme Rivory, Mathieu Pioche

**Affiliations:** 1 Department of Systems Medicine, Gastroenterology and Endoscopy Unit, University of Rome Tor Vergata, Rome, Italy; 2 Department of Endoscopy and Hepatogastroenterology, Pavillon L, Edouard Herriot Hospital, Lyon, France; 3 Department of Endoscopy and Hepatogastroenterology, Clermont-Ferrand University Hospital, Université Clermont Auvergne, Clermont-Ferrand, France; 4 Department of Gynecology, Clermont-Ferrand University Hospital, Université Clermont Auvergne, Clermont-Ferrand, France

A rectovaginal fistula is a rare condition and a therapeutic challenge 1 . Endoscopic closure requires combined approaches with both mucosal ablation and mechanical closure of the fistula 2 . As reported in a recent multicenter study, the novel “fistula endoscopic submucosal dissection with clip closure” (FESDC) strategy is safe and feasible for endoscopic closure of gastrointestinal fistulas 3 ; however, in our population, the technical success does not exceed 70 % in naïve fistulas. We previously reported the optimal results of a new closure device called the “mucosal adaptative ring to close endoscopic artificial ulcer” (MARCEAU) in endoscopic leak closure at the ulcer bed after endoscopic submucosal dissection (ESD) and persistent gastrocutaneous fistulas after percutaneous endoscopic gastrotomy 4 5 . This strategy has not previously been used for gynecological fistulas.

We herein report the case of a 31-year-old woman referred for a rectovaginal fistula after a colonic resection for endometriosis, which had caused severe infections (chorioamniotitis leading to miscarriage). A sigmoidoscopy was performed, and the fistulous orifice appeared in the rectum, with passage of air and blue in the vagina ( Video 1 ). ESD of the surrounding mucosa was performed to facilitate fistula closure and the MARCEAU device was used to close the fistula. This device is made from anti-return sutures (VLOC; Medtronic, USA) with a loop that can be pulled, thereby closing the loop progressively ( Fig. 1 ). The system was fixed to two edges of the ulcer by clips. Multiple clips were then placed side by side capturing the sealing device. Finally, all of the clips were closed up to each other by tightening the device with a forceps ( Fig. 2 ). An MRI performed 3 months after the procedure confirmed the closure of the fistula.

**Video 1** Rectovaginal fistula treated with MARCEAU device.


**Fig. 1:**
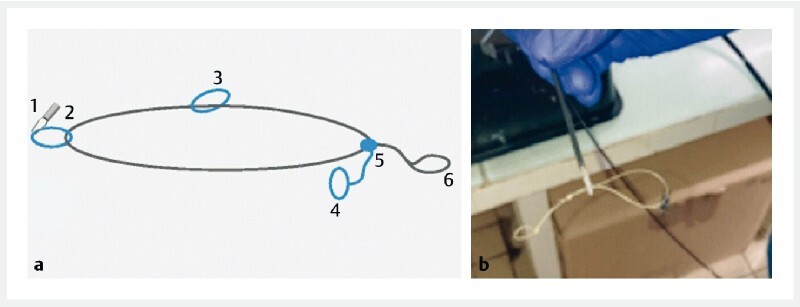
The MARCEAU device is shown in: **a** a drawing (1, edge loop; 2, hemoclip; 3, additional fixation loop; 4, fixed point for one edge; 5,6, tightening wire with the loop to grasp and pull); **b** a photograph.

**Fig. 2:**
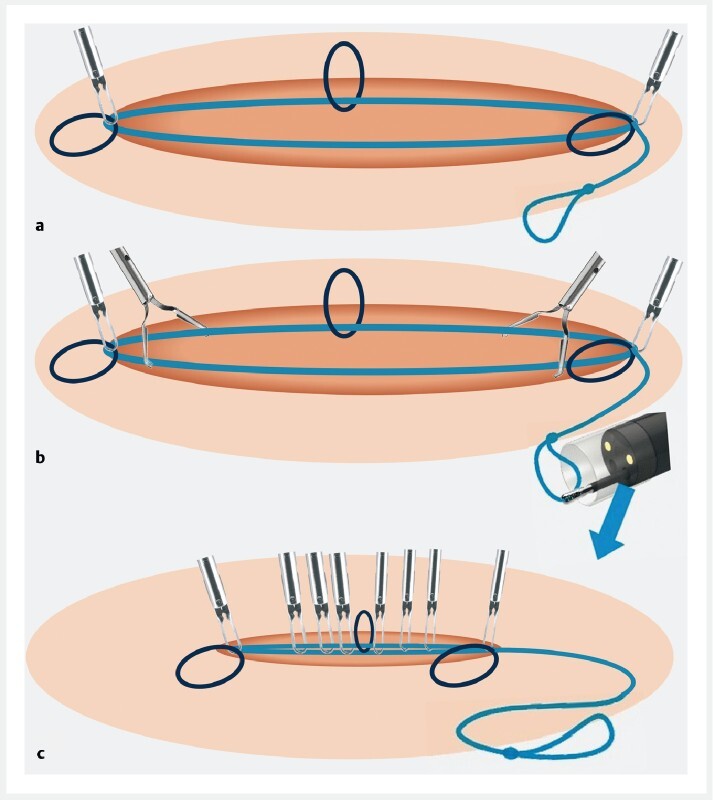
Schematic view of the “mucosal adaptive ring to close an endoscopic artificial ulcer” (MARCEAU) device showing: **a** fixation of the device at the edge of the ulcer; **b** clipping along the ulcer; **c** closure of the ulcer by tightening the device with a forceps.

This technique seems attractive in challenging situations, such as gynecological fistulas, to ensure a firm and sustained closure of the leak. The low cost and ease of use of this device combined with lower risks than surgery could allow more widespread use.

Endoscopy_UCTN_Code_TTT_1AQ_2AG

## Notes

Endoscopy E-Videos

https://eref.thieme.de/e-videos
*E-Videos* is an open access online section of the journal *Endoscopy* , reporting on interesting cases and new techniques in gastroenterological endoscopy. All papers include a high-quality video and are published with a Creative Commons CC-BY license. Endoscopy E-Videos qualify for HINARI discounts and waivers and eligibility is automatically checked during the submission process. We grant 100% waivers to articles whose corresponding authors are based in Group A countries and 50% waivers to those who are based in Group B countries as classified by Research4Life (see: https://www.research4life.org/access/eligibility/ ). This section has its own submission website at https://mc.manuscriptcentral.com/e-videos
